# Mindfulness-Based Interventions for People Diagnosed with a Current Episode of an Anxiety or Depressive Disorder: A Meta-Analysis of Randomised Controlled Trials

**DOI:** 10.1371/journal.pone.0096110

**Published:** 2014-04-24

**Authors:** Clara Strauss, Kate Cavanagh, Annie Oliver, Danelle Pettman

**Affiliations:** 1 School of Psychology, University of Sussex, Falmer, United Kingdom; 2 Sussex Partnership NHS Foundation Trust, Brighton, United Kingdom; Federal University of Rio de Janeiro, Brazil

## Abstract

**Objective:**

Mindfulness-based interventions (MBIs) can reduce risk of depressive relapse for people with a history of recurrent depression who are currently well. However, the cognitive, affective and motivational features of depression and anxiety might render MBIs ineffective for people experiencing current symptoms. This paper presents a meta-analysis of randomised controlled trials (RCTs) of MBIs where participants met diagnostic criteria for a current episode of an anxiety or depressive disorder.

**Method:**

Post-intervention between-group Hedges *g* effect sizes were calculated using a random effects model. Moderator analyses of primary diagnosis, intervention type and control condition were conducted and publication bias was assessed.

**Results:**

Twelve studies met inclusion criteria (n = 578). There were significant post-intervention between-group benefits of MBIs relative to control conditions on primary symptom severity (Hedges *g* = −0.59, 95% CI = −0.12 to −1.06). Effects were demonstrated for depressive symptom severity (Hedges *g* = −0.73, 95% CI = −0.09 to −1.36), but not for anxiety symptom severity (Hedges *g* = −0.55, 95% CI = 0.09 to −1.18), for RCTs with an inactive control (Hedges *g* = −1.03, 95% CI = −0.40 to −1.66), but not where there was an active control (Hedges *g* = 0.03, 95% CI = 0.54 to −0.48) and effects were found for MBCT (Hedges *g* = −0.39, 95% CI = −0.15 to −0.63) but not for MBSR (Hedges *g* = −0.75, 95% CI = 0.31 to −1.81).

**Conclusions:**

This is the first meta-analysis of RCTs of MBIs where all studies included only participants who were diagnosed with a current episode of a depressive or anxiety disorder. Effects of MBIs on primary symptom severity were found for people with a current depressive disorder and it is recommended that MBIs might be considered as an intervention for this population.

## Introduction

Mindfulness refers to a state of consciousness that is characterised by the self-regulation of attention towards present-moment experiences coupled with an accepting, non-judgemental stance towards these experiences [1]. Mindfulness-based interventions (MBIs) are usually brief interventions (typically eight sessions) delivered in a group setting and which incorporate mindfulness meditation practice and principles. Mindfulness-Based Stress Reduction (MBSR) [2] and Mindfulness-Based Cognitive Therapy (MBCT) [3] are the most widely evaluated and available approaches. MBSR was developed in the late 1970s and, with its emphasis on stress reduction and improving wellbeing, has been applied widely across physical health, mental health and non-clinical populations. MBCT was developed in the 1990s and integrates MBSR with elements from cognitive therapy for depression. It was designed originally as a relapse prevention intervention for people with a history of recurrent depression, although in recent years MBCT has been extended to people with current diagnoses of depressive and anxiety disorders.

There is evidence that MBCT approximately halves the risk of relapse in comparison to standard care for people who are currently well but who have experienced at least three prior episodes of depression [4], [5] and is comparable to anti-depressant medication in reducing risk of relapse [6]. Because of promising findings such as these, MBCT is now recommended in national guidelines as a treatment choice for relapse prevention in recurrent depression [7] and implementation of these recommendations is underway [8]. Due to such promising findings there has been a move to extend the reach of MBIs to people experiencing a current episode or an anxiety or depressive disorder [9], [10], [11], [12]. However, neither MBSR nor MBCT were developed for people experiencing an acute episode of depression or anxiety [3], [13], [14]. Limited research has evaluated the effectiveness of these interventions within this currently distressed population and there are good reasons to be cautious about extending the reach of MBIs in this way.

In contrast to populations with a history of depression who are not currently distressed, there are at least three reasons to suspect that standard MBIs may not be of benefit to populations currently meeting diagnostic criteria for an episode of a depressive or anxiety disorder. First, MBIs invite participants to bring their full awareness to current experiences. For people with a current episode of an anxiety or depressive disorder their current experiences are likely to include aversive automatic thoughts [15], [16] and unpleasant feelings of low mood or anxious arousal, which may be difficult for the individual to attend to or accept. A brief MBI of only eight sessions may be insufficient to enable people to learn to attend to and accept such experiences. Whilst MBCT emphasises decentring from unpleasant experiences, rather than changing the content of experiences, cognitive behaviour therapy (CBT) aims to change the content of negative thoughts and beliefs and this may hold more intuitive appeal for people who are currently depressed.

Second, cognitive processes common in anxiety and depressive disorders may run counter to learning mindfulness [18]. Rumination [19], [20], worry [21] and attentional biases [22] are characteristic of depression and anxiety and mean that people with a current episode of an anxiety or depressive disorder are likely to become preoccupied by negative thoughts and feelings. Attempting to distract from or avoid unpleasant experiences is also common in depression and anxiety [23], [24]. Mindfulness represents a different way of responding to experience by being aware of experience (so not avoiding or distracting from it) without attaching to it (so without perseverating) and this may be a difficult skill to learn during a brief MBI that was not designed for these populations.

Finally, there are motivational and concentration difficulties that may present a challenge for people with a current episode of an anxiety or depressive disorder to engaging in mindfulness practice. Learning to self-regulate attention is seen as one of the cornerstones of MBIs [1] however, regulating attention can be difficult for people experiencing anxiety or depression [9], [25], [26]. Brief MBIs may not be sufficient to enable people experiencing a current episode of an anxiety or depressive disorder to learn to regulate their attention more effectively.

Whilst a number of meta-analyses have explored the effectiveness of MBIs in relation to symptoms of anxiety and depression [27], [28], [29], [30], [31], [32], none directly address the effectiveness of MBIs for people experiencing a current anxiety or depressive episode in comparison to control conditions. Answering this question will help to inform the appropriate implementation of MBIs in routine clinical care.

The current meta-analysis tests the effectiveness of MBIs in randomised controlled trials where all participants were diagnosed with a current episode of an anxiety or depressive disorder. This is operationalized as all participants in a study meeting diagnostic criteria for a current episode of a DSM-IV (or later version) or ICD-10 depressive or anxiety disorder [33], [34]. MBIs in this study were limited to interventions where mindfulness was core to the intervention, that included mindfulness practice in each therapy session and where daily mindfulness practice is recommended. These criteria excluded relapse prevention trials where participants have a history of depression but who are not experiencing a full current episode and trials where mindfulness practice is not foregrounded.

This meta-analysis addressed the important question of whether the conclusions about the effectiveness of MBIs can be extended to people experiencing a current episode or an anxiety or depressive disorder based on findings from RCTs. The primary outcome was symptom severity for the target clinical problem. Secondary outcomes of anxiety and depression symptom severity (irrespective of diagnosis) were used. Moderator analyses looking at primary diagnosis (anxiety or depressive disorder), control condition (inactive or active) and intervention type (MBCT or MBSR) were planned in the event of significant outcome heterogeneity.

## Method

No published protocol has been published for this meta-analysis. The PRISMA guidelines were adhered to [35]. See Checklist S1 for details (supporting information).

### Search Strategy

Titles and abstracts from the following databases were searched: MEDLINE, Web of Science, Scopus, ProQuest and PsycINFO for published or unpublished studies from the first available year of publishing until 4 July 2013. Reference sections of identified papers were searched manually. The following search terms were used: [(mindfulness or MBCT or MBSR) combined with (anxi* or depress* or OCD or “obsessive compulsive” or “post-traumatic stress disorder” or PTSD or agoraphobia or “panic disorder” or “acute stress disorder” or “acute stress reaction” or phobi*) combined with (random* or RCT)].

In order to conduct a replicable search for unpublished data, three leading clinical trial registers (www.clinicaltrialsregister.eu, clinicaltrials.gov and www.controlled-trials.com/isrctn) were searched to identify completed clinical trials of MBIs that had not been published. The trial registers were searched with the term ‘mindfulness’ (multiple search terms were not possible) with no restrictions placed on the search. All identified research team members from relevant clinical trials were contacted by email for details of their findings. In the event of failing to respond to email requests a further two emails were sent.

### Inclusion and Exclusion Criteria

Inclusion criteria were: (1) designs were Randomized Control Trials; (2) participants aged 18 years or over; (3) mindfulness was a core part of the MBI with mindfulness practice in each therapy session and daily practice encouraged between sessions; (4) studies included a psychometrically reliable and valid outcome measure of depression or anxiety; and (5) data was presented for participants who met full diagnostic criteria for a current episode of a DSM-IV (or later version) or ICD-10 anxiety or depressive disorder. Specifically, participants were required to meet full diagnostic criteria for a DSM-IV depressive disorder (Major Depressive Disorder not in full or partial remission) or anxiety disorder (Generalised Anxiety Disorder, Panic Disorder, Agoraphobia, Specific Phobia, Social Phobia, Obsessive Compulsive Disorder, Post-Traumatic Stress Disorder or Acute Stress Disorder) or an ICD-10 depressive episode or depressive disorder or anxiety disorder (Phobic Anxiety Disorder, Agoraphobia, Social Phobia, Specific Phobia, Panic Disorder, Generalised Anxiety Disorder, Obsessive-Compulsive Disorder, Acute Stress Reaction or Post-Traumatic Stress Disorder). Hypochondriasis (health anxiety) was also an inclusion criterion given the central role that anxiety plays in the disorder [33], [34].

Exclusion criteria were: (1) participants had marked cognitive impairment (e.g. learning disability or brain injury); (2) participants were currently engaged in substance misuse; (4) MBI was not delivered in a group format; (5) MBI was not delivered in-person (e.g. self-help); and (6) studies not in the English language. Duplicate data was also excluded if the same outcomes were fully or partially reported in another study. When this occurred, the study with the larger sample size was retained.

### Data Entry and Analysis

Means, standard deviations (sd) and number of participants for the primary symptom measure and for measures of depression and anxiety were entered into Review Manager version 5.2 (RevMan 5.2 [36]. Where available, intention-to-treat (ITT) data were entered, where only completer data were available these were used (see Table 1 for details). Post-intervention between-group effect sizes were calculated using a random effects model (as this allows generalisation of findings beyond the set of included studies) and the following formula for Hedges *g* was used to calculate the effect size for each study:
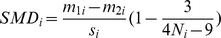
where;

**Table 1:** Details of included studies.

Study	Diagnostic Criteria (age: mean and sd/years) [psychotropic mediation/%]	MBI (baseline n) Control (baseline n)	Primary Outcome Measure	Depression Outcome Measure	Anxiety Outcome Measure	Attrition from MBI (typically defined as <50% sessions)	Data Type	Jadad Score (0–5)
Arch et al. (2013)	Participants met DSM-IV criteria for an anxiety disorder based on the MINI interview. (m = 45.9 yrs, sd = 13.68) [84.62%]	MBSR (45) CBT (60)	MASQ-AA	BDI-II	MASQ-Anxious Arousal Scale	38%	Completer	3
Asmaee Majid et al. (2012)	Participants met DSM-IV criteria for generalised anxiety disorder on a SCID interview. (m = 32.19 yrs, sd = 2.21) [medication use not reported]	MBSR (16) Control (15)	PSWQ	BDI-II	PSWQ	<7% (not reported by group)	Completer	2
Chiesa et al. (2012)	Participants met DSM-IV criteria for major depressive disorder and score 8 or above on the HAMD. (All 18+ yrs - specific age information not provided) [100%]	MBCT (9) Group Psychoed (9)	HAMD	HAMD	BAI	11%	ITT	3
Jazaieri et al. (2012)	Participants met criteria for a principal DSM-IV diagnosis of generalised social anxiety disorder based on an ADIS-IV-L interview. (m = 32.8 yrs, sd = 8.4) [21.4%]	MBSR (31) Aerobic Exercise (25)	LSAS-SR	BDI-II	LSAS-SR	16%	Completer	3
Kearney et al. (2013)	Participants were veterans meeting DSM-IV PTSD diagnosis confirmed through case notes. (MBSR: m = 52 yrs, sd = 13.4 TAU: m = 52 yrs, sd = 11.7) [>64% (multiple medications reported)]	MBCT (25) TAU (22)	PCL	PHQ-9	PCL	8% (study drop out)	ITT	5
Koszycki et al. (2007)	Participants met DSM-IV social anxiety disorder criteria assessed through a MINI interview and they had an LSAS total score over of 50 or more. (MBSR: m = 38.9 yrs, sd = 15.7 (CBT: m = 37.6 yrs, sd = 11.1) [MBSR = 30.8%; GCBT = 25.9%]	MBSR (26) Group CBT (27)	LSAS-A LSAA-C (composite)	BDI-II	LSAS-A LSAA-C (composite)	15%	ITT	2
Manicavasagar et al. (2012)	Participants met DSM-IV criteria for major depressive disorder confirmed through a CIDI interview and they had a BDI-II score of 20 or above. (MBCT: m = 47 yrs, sd = 13.8 CBT: m = 45 yrs, sd = 12.9) [60%]	MBCT (30) Group CBT (39)	na	BDI-II	na	37%	Completer	2
McManus et al. (2012)	Participants met DSM-IV criteria for a primary diagnosis of hypochondriasis assessed through SCID interview. (MBCT: m = 41.28 yrs, sd = 11.98; TAU: m = 43.92 yrs, sd = 10.98) [MBCT = 44.4%; TAU = 39.5%]	MBCT (36) TAU (38)	SHAI	BDI-II	SHAI	11%	ITT	3
Piet et al. (2010)	Participants met criteria for a DSM-IV diagnosis for social anxiety disorder based on ADIS-IV-L interview. (MBCT: m = 21.6 yrs, sd = 2.84 GCBT: m = 22.1 yrs, sd = 2.54) [MBCT = 14%; GCBT = 42%]	MBCT (14) Group CBT (11)	LSAS	BDI-II	LSAS	21%	ITT	3
Strauss et al. (2012)	Participants had a consultant psychiatrist confirmed DSM-IV diagnosis of chronic depression and a BDI-II score of 20 or more. (m = 43 yrs, sd = 10.6) [88%]	PBCT (14) TAU (14)	BDI-II	BDI-II	na	21%	ITT	3
Van Aalderen et al. (2012)	Participants had diagnosis of a current DSM-IV major depressive episode assessed through SCID interview and a confirmed DSM-IV recurrent depressive disorder. (MBCT: m = 47.3 yrs, sd = 11.5 TAU: m = 47.7 yrs; sd = 11.1) [MBCT = 52%; TAU = 47%]	MBCT (34) TAU (35)	BDI-II	BDI-II	na	8%	Completer	3
Vøllestad et al. (2011)	Participants met DSM-IV criteria for panic disorder, agoraphobia, social anxiety disorder or generalised anxiety disorder assessed through the MINI. (m = 42.5 yrs, sd = 11.3) [27.6%]	MBSR (39) Wait-list (37)	STAI	BDI-II	STAI	21%	ITT	2

CBT = Cognitive Behavioural Therapy, MBCT = Mindfulness-based Cognitive Therapy, MBSR = Mindfulness-based Stress reduction, PBCT = Person-based Cognitive Therapy.

HA = Health Anxiety, SAD = Social Anxiety Disorder, MDD = Major Depressive Disorder, OCD = Obsessive Compulsive Disorder, PTSD = Post-Traumatic Stress Disorder, PD = Panic Disorder, AG = Agoraphobia, GAD = Generalised Anxiety Disorder.

ADIS-IV-L = Anxiety Disorders Interview Schedule for DSM-IV-Lifetime version (Di Nardo, Brown, & Barlow, 1994), BDI-II = Beck Depression Inventories second edition (BDI–II: Beck, Steer & Brown, 1996), CIDI = Composite International Diagnostic Interview (WHO, 1997), PHQ-9 = Patient Health Questionnaire-9 for depression (PHQ-9; Kroenke, Spitzer, & Williams, 2001), HAMD = Hamilton Rating Scale for Depression (Hamilton, 1960), FFMQ = Five Facet Mindfulness Questionnaire (FFMQ: Baer et al., 2008), KIMS = Kentucky Inventory of Mindfulness (Baer et al, 2004), LSAS-SR = Liebowitz Social Anxiety Scale-Self-Report (Fresco et al., 2001), LSAS-avoidance subscale (Liebowitz, 1987), LSAS-fear subscale (Liebowitz, 1987), MAAS = Mindfulness Attention and Awareness Scale (Brown & Ryan, 2003), MASQ-AA = Mini Mood and Anxiety Symptom Questionnaire (Casillas & Clark, 2000), PSWQ = Penn State Worry Questionnaire (Meyer et al, 1990), SHAI = Short Health Anxiety Inventory (Salkovskis et al.,2002), MINI = Mini International Neuropsychiatric Interview (Sheehan et al, 1998), SCID = Structured Clinical Interview for DSM–IV–TR Axis I disorders (First, Gibbon, Spitzer, & Williams, 2002, STAI = Spielberger State Trait Anxiety Inventory (Spielberger et al,1983), SMQ = Southampton Mindfulness Questionnaire (Chadwick et al., 2008), Y-BOCS = Yale Brown Obsessive Compulsive Scale (Goodman et al, 2006).



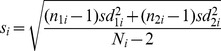



SMD_i_ = ‘standardised mean difference’; m_1i_ = post-intervention mean on chosen outcome measure for group 1; m_2i_ = post-intervention mean on chosen outcome measure for group 2; N_i_ = total number of participants (across both conditions); s_i_ = pooled standard deviation; n_1i_ = number of participants in group 1; n_2i_ = number of participants in group 2; sd_1i_ = standard deviation of the post-intervention mean for group 1; sd_2i_ = standard deviation of the post-intervention mean for group 2.

In essence, Hedges *g* effect size is the between-group difference on the post-intervention mean scores for the chosen outcome measure, divided by the pooled standard deviation (roughly speaking, the average standard deviation of the two groups) and then multiplied by a figure that adjusts the effect size to take account of small sample sizes. This formula gives larger effect sizes as the difference between the post-intervention means of the two groups increase. By Cohen’s convention a small effect size is 0.2, a medium effect size is 0.5 and a large effect size is 0.8.

Forest plots of post-intervention between-group effect sizes were produced for each of the three outcome variables. To address publication bias Rosenthal’s Fail-Safe N [37] was computed to estimate the number of equal sample size unpublished studies of zero effect that would be needed to reduce the mean effect size to being non-significant in addition to producing funnel plots showing study effect sizes against their standard error. A funnel plot that shows points evenly distributed around the mean effect size (shown as a vertical line) and forming a funnel shape indicates that publication bias may not be present. Publication bias is suggested if the funnel shape is distorted to show a disproportionate number of studies with larger standard errors showing larger than expected effect sizes. This is based on the assumption that large-scale, funded RCTs tend to publish their findings (with large scale studies produced smaller standard errors) whereas small-scale studies may fail to publish non-significant or negative findings.

In order to assess the extent to which effect sizes were significantly different from each other heterogeneity was assessed using chi-square. A significant chi-square value indicates heterogeneity and that the studies cannot be considered to have recruited from the same population. In this case, possible reasons for heterogeneity can be explored. In order to do this moderator analyses were planned to explore effects as a function of control group (active or inactive), presenting problem (anxiety or depressive disorder) and MBI-type (e.g. MBCT or MBSR).

The Jadad rating scale [38] was used to establish the quality of each study using the following criteria: (a) the study was described as randomized; (b) the method of randomization was appropriate; (c) the study was described as double-blind, (d) the method of double blinding was appropriate; and, (e) the study includes information about all drop-outs and withdrawals. Each criterion was awarded 1 point with a maximum score of 5.

## Results

**Figure 1:**
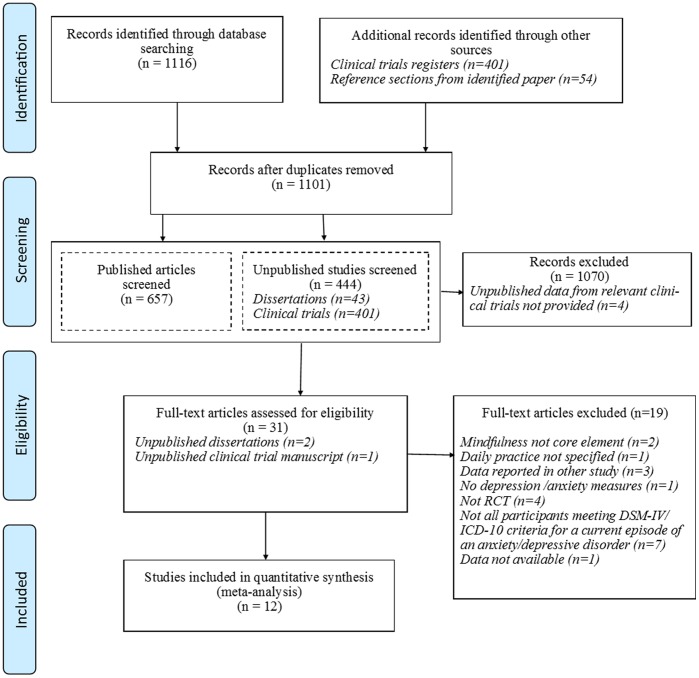
PRISMA (2009) Flow Diagram.

After duplicates were removed 657 published articles and 43 unpublished dissertations were identified, 30 records remained after screening abstracts (see Figure 1 for full details). The full text of these papers were reviewed and inclusion and exclusion criteria applied which resulted in 12 studies included in the meta-analysis [39], [40], [41], [42], [43], [44], [45], [46], [47], [48], [49], [50]. See Figure 1 for a flow diagram detailing the search and Table 1 for details of the 12 studies.

The search of clinical trial registers produced a total of 401 records. Titles and research protocols of trials which had closed to recruitment were screened and the meta-analysis inclusion/exclusion criteria were applied. Five trials were identified as possible candidates following this screen. All named members of the research teams on these trials were contacted by email for information about the studies, however no data meeting our search criteria were made available.

### Participant Characteristics

There were a total of 578 participants across the 12 studies. All participants had a DSM-IV confirmed diagnosis of a major depressive disorder or an anxiety disorder. The DSM-IV diagnosis of participants was major depressive disorder (4 studies, total n = 160) or an anxiety disorder (8 studies, total n = 418: social anxiety disorder (3 studies, total n = 120), generalised anxiety disorder (1 study, total n = 31), PTSD (1 study, total n = 47), or health anxiety (1 study, total n = 74). Two studies included participants with a range of DSM-IV anxiety disorders (n = 146).

Table 1 shows that the mean age of participants were typically in the 30s or 40s with mean ages ranging from 21 years to 52 years. Whilst most studies did not report age ranges, the standard deviation of ages suggest that most, if not all, participants were of working age (18–65 years). All but one study reported use of psychotropic medication. This ranged from 14% in one study to 100% in another study.

### Mindfulness-Based Interventions

Mindfulness-based interventions were Mindfulness-Based Cognitive Therapy (MBCT = 6), Mindfulness-Based Stress Reduction (MBSR = 5) and Person-Based Cognitive Therapy (PBCT = 1). MBCT and MBSR are group approaches consisting of eight 2 to 3 hour weekly sessions plus one whole day session. They involve a range of mindfulness practices which range between 3 and 40 minutes in length, with more than one practice per session. Daily mindfulness practice between sessions is encouraged and supported through audio recordings. In addition there is in-session discussion of what was learned during mindfulness practice. PBCT for depression involves twelve 90-minute sessions. There are two mindfulness practices in each session (a 5 minute practice and a 10 minute practice) along with Socratic discussion of what was learned and daily mindfulness practice is encouraged and supported through mindfulness practice audio recordings. Both MBCT and PBCT include elements of cognitive therapy with a greater emphasis placed on cognitive therapy in PBCT than in MBCT.

### Control Conditions

There were five active control conditions (group CBT = 4, group psychoeducation = 1) and seven inactive control conditions (TAU = 5, wait-list = 1, aerobic exercise = 1).

### Attrition

There was wide variability in the number of participants dropping out from MBI with attrition ranging from 8 percent to 38 percent (median attrition = 15.5%).

### Meta-Analysis Findings

#### Effect of MBI on primary symptom severity

**Figure 2:**
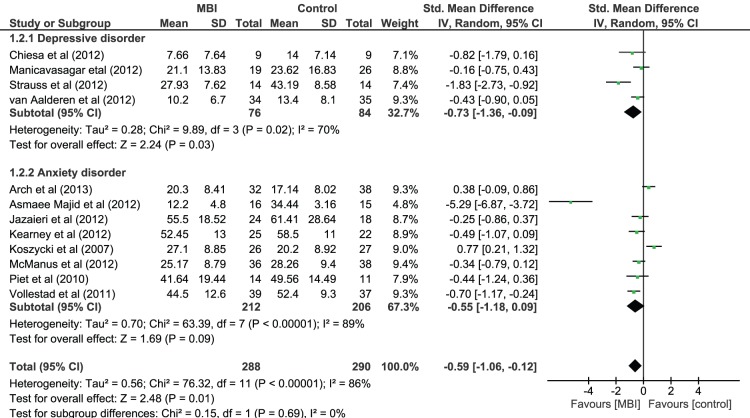
Forest Plot of MBIs in comparison to control conditions on severity of primary symptom for people with depressive disorder and anxiety disorder diagnoses.

A random effects model (see Figure 2) showed there was a post-intervention between-group difference in favour of MBI on primary symptom severity with a medium effect size (Hedges *g* = −0.59, 95% CI = −1.06 to −0.12) that was statistically significant (*z*(11) = 2.48, *p* = 0.01). Heterogeneity was significant (*χ^2^*(11) = 76.32, *p*<.001) and so moderator analyses were performed on primary diagnosis (depressive or anxiety disorder), type of control condition (active or inactive) and type of intervention.

#### Primary symptom effect size as function of primary diagnosis

Moderator analysis showed no significant differences in primary symptom severity between those studies targeting depressive disorders and those studies targeting anxiety disorders (*χ^2^*(1) = 0.15, *p* = 0.69). However, analyses within subgroups (see Figure 2) showed that whilst there were significant post-intervention between-group differences for people diagnosed with a depressive disorder with a large effect size in favour of MBI on primary symptom severity (Hedges *g* = −0.73, 95% CI = −1.36 to −0.09, *z*(3) = 2.24, *p* = .03) effects for anxiety disorders were non-significant (Hedges *g* = −0.55, 95% CI = −1.18 to 0.09, *z*(7) = 1.69, *p* = .09).

#### Primary symptom effect size as function of control condition type

**Figure 3:**
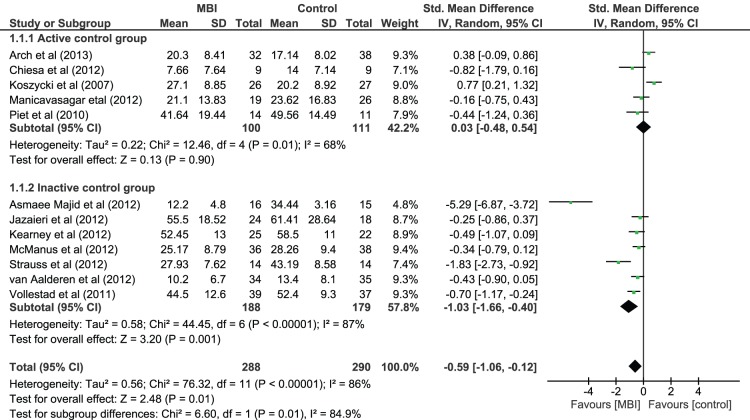
Forest Plot of the effect of MBIs in comparison to control conditions on primary symptom severity by control condition type (active versus inactive) for people with depressive disorder and anxiety disorder diagnoses.

Figure 3 shows that moderator analysis found a significant difference between studies with active and inactive control conditions (*χ^2^*(1) = 6.60, *p* = .001). Whilst MBI outperformed inactive control conditions with a large effect size when looking at primary symptom severity (Hedges *g* = −1.03, 95% CI = −1.66 to −0.40, *z*(6) = 3.20, *p* = .001) MBI was not significantly different than active control conditions (Hedges *g* = 0.03, 95% CI = −0.48 to 0.54, *z*(4) = 0.13, *p* = .90). This suggests that the effect of MBIs on primary symptom severity varied as a function of control condition with MBIs being more effective than inactive control conditions but not more effective than active control conditions.

#### Primary symptom effect size as function of intervention type (MBCT or MBSR)

**Figure 4:**
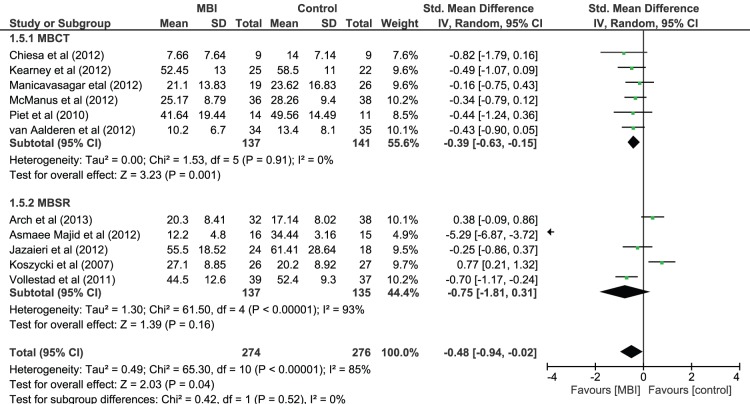
Forest Plot of the effect of MBIs in comparison to control conditions on primary symptom severity by intervention type (MBCT versus MBSR) for people with depressive disorder and anxiety disorder diagnoses.

Moderator analysis was also conducted on the effect of intervention type on primary symptom severity for studies MBCT and MBSR (see Figure 4). The single PBCT study was not included in this analysis. This analysis showed no significant differences between MBI subgroups (*χ^2^*(1) = 0.42, *p* = .52). However, when looking at these subgroups separately there was no significant effect of MBSR on primary symptom severity (Hedges *g* = 0.75, 95% CI = −1.81 to 0.31, *z*(4) = 1.39, p = .16) but there was a significant effect of MBCT with a small to medium effect size (Hedges *g* = 0.39, 95% CI = −0.63 to −0.15, *z*(5) = 3.23, *p*<.01).

#### Effect of MBI on depressive and anxiety symptom severity (irrespective of diagnosis)

**Figure 5:**
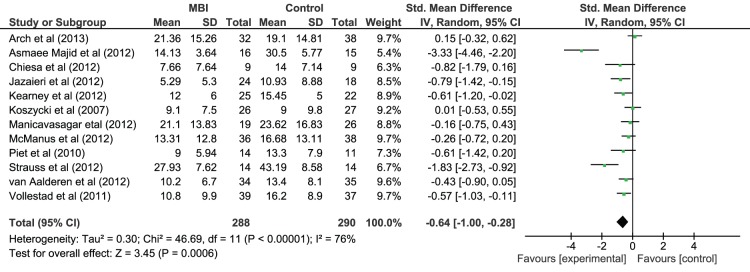
Forest Plot of the effect of MBIs in comparison to control conditions on depressive symptom severity for people with depressive disorder and anxiety disorder diagnoses.

**Figure 6:**
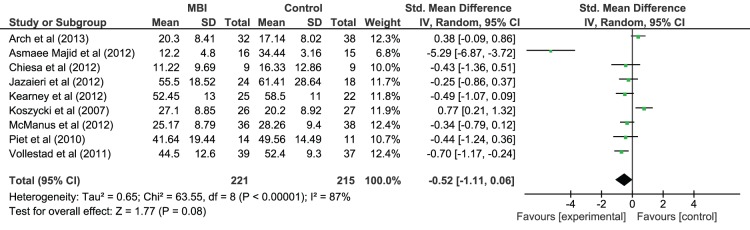
Forest Plot of the effect of MBIs in comparison to control conditions on anxiety symptom severity for people with depressive disorder and anxiety disorder diagnoses.

Given the high co-morbidity between anxiety and depression [51], meta-analyses were also conducted on depressive and anxiety symptom severity, irrespective of primary diagnosis (see Figure 5 and Figure 6 respectively). All the studies included a measure of depressive symptom severity. There was a post-intervention between-group effect in favour of MBI on depressive symptom severity with a medium effect size (Hedges *g* = −0.64, 95% CI = −1.00 to −0.28) that was statistically significant (*z*(11) = 3.45, *p*<.001) but effect sizes were heterogeneous (*χ^2^*(11) = 46.69, *p*<.001). Nine studies included a measure of anxiety symptom severity and there was a non-significant post-MBI between-group difference in anxiety symptom severity (Hedges *g* = −0.52, 95% CI = −1.11 to 0.06, *z*(8) = 1.77, *p* = .08) and heterogeneous effect sizes (*χ^2^*(8) = 63.55, *p*<.001). These results suggest that MBIs had an effect of depressive symptom severity relative to control conditions but did not have a significant effect on anxiety symptom severity.

#### Publication bias

**Figure 7:**
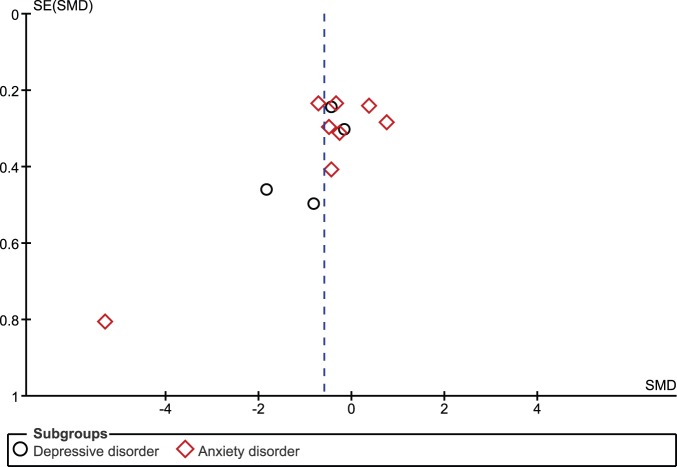
Funnel plot of effect sizes by standard error for primary symptom severity.

In terms of publication bias, the funnel plot for primary symptom severity (see Figure 7) suggests a slight bias towards publishing small sample size studies with findings in favour of MBI. This is shown by the disproportionate number of small studies (shown towards the bottom of the figure) with larger effect sizes than would be suggested by the overall effect. However, Rosenthal’s Fail-Safe N analyses found that an additional 264 studies showing no intervention effect would be needed to reduce the overall effect size on primary symptom severity to being non-significant. This indicates that whilst a publication bias may be present, a substantial number of unpublished studies would need to exist to render these effects non-significant.

#### Study quality and effect sizes

Jadad scores for studies ranged from 2 to 5 (mean = 2.83, sd = 0.83). The correlation between Jadad ratings and study effect sizes was non-significant for primary symptom severity (*r*(12) = −.20, *p* = .54), depressive symptom severity (*r*(12) = −.14, *p* = .66) and for anxiety symptom severity (*r*(8) = .26, *p* = .53). This shows no significant relationship between study quality and study effect size.

## Discussion

This meta-analysis tested the effectiveness of MBIs for people diagnosed with a current episode of a depressive or anxiety disorder in comparison to control conditions. This analysis was restricted to randomised controlled trials where all participants were confirmed as meeting diagnostic criteria for a current episode of a depressive or anxiety disorder and where intervention participants were assigned to MBIs that foregrounded mindfulness principles and practice as a core feature of the intervention. We found that MBIs, in comparison to control conditions, resulted in significantly lower levels of symptom severity for the primary problem with a medium between-group effect size. This suggests that, despite the cautions outlined in the Introduction, these interventions are associated with significant primary symptom benefits for these populations.

### Findings in Context

Published meta-analyses of MBIs for mental health conditions are limited for three reasons. Firstly, some are limited in their methodological rigor by foregrounding pre-post effect sizes and not providing a robust comparison to control conditions [28], [30] or by reporting between-group effect sizes but including non-randomised trials [29], [32]. Second, some are limited by having overly broad inclusion criteria for the type of intervention, such as including interventions that do not foreground mindfulness practice [29], [32], calling into question their relevance when addressing the effectiveness of MBIs. Finally, some are limited by participant characteristics with some existing meta-analysis not restricting eligibility to only those studies with participants meeting diagnostic criteria for a current anxiety or depressive disorder [27], [29], [31] whereas some focus on specific disorders [29], [32] rather than considering anxiety and depressive disorders together which is arguably a false dichotomy given the high co-morbidity between depressive and anxiety disorders [51]. This is the first published meta-analysis of RCTs that demonstrates that people experiencing a current episode of an anxiety or depressive disorder can benefit from MBIs. However, effect sizes were heterogeneous and moderator analyses revealed a more nuanced picture.

### Effects on Depression

There were significant benefits on primary symptom severity of MBIs relative to control conditions for people with a primary depressive disorder diagnosis. There were also significant benefits of MBI on depressive symptom severity across the studies, irrespective of primary presenting problem. Effects of MBI on depressive symptom severity replicates and extends findings from previous meta-analyses [28], [29]. Findings for effects on depression are in line with effect sizes reported in a recent meta-analysis [29] where a post-intervention between-group effect size of −0.53 was found on depressive symptom severity, which is somewhat smaller than the effect size of −0.73 found in the current meta-analysis. This previous meta-analysis however was not restricted to studies where participants were confirmed as meeting diagnostic criteria for major depressive disorder. Therefore, the finding from the current meta-analysis makes an important and novel contribution to the clinical literature as it shows that people experiencing a current episode of major depressive disorder can gain symptom benefit from MBIs.

We suggested earlier that certain features of depression could present a barrier to people engaging in and benefitting from MBIs. The aversive content of depressive thoughts and feelings [15] could present a challenge to engaging in mindfulness practice, the process of rumination, common in depression [20], runs counter to mindfulness and the attentional and motivational features of depression [22] could make it difficult to self-regulate attention [1] and commit to regular mindful practice. Despite these potential barriers we found that people experiencing a current episode of a depressive disorder could benefit from MBIs.

### Effects on Anxiety

Although moderator analyses did not show that the effects of MBIs on primary symptom severity varied as a function of primary problem, the effects on anxiety symptom severity were not statistically significant either when just looking at those people with a confirmed anxiety disorder diagnosis or when looking at anxiety outcomes across the full-range of studies. Although the mean effect size for the effect of MBIs on anxiety symptom severity was in the moderate range (Hedges *g* = −0.52), the 95% confidence interval for this effect crossed zero (−1.11 to 0.06). Moreover, one of the studies included in this analysis had a low Jadad rating [40] and produced an unusually large effect size of −5.29. If this study is removed the mean effect size becomes small (Hedges *g* = −0.17; 95% confidence interval: −0.54 to 0.21). Overall, we suggest that caution should be applied in offering MBIs for populations with anxiety disorders or where anxiety symptom severity is a target. However, the failure to find an effect on anxiety symptom severity could be due to a lack of power in this analysis and further studies are needed before we can draw definitive conclusions about the beneficial effects of MBIs for people with a current anxiety disorder.

These findings appear to be in contrast to a recent meta-analysis of mindfulness and acceptance-based interventions [32] where significant between-group effects were reported for controlled trials on anxiety symptom severity for people with a confirmed anxiety disorder. However, the between-group analysis in that paper included only five studies, including two studies, with the largest effect sizes, that were not MBIs. The current meta-analysis used a strict definition of MBIs in order to isolate, as well as possible, the effect of mindfulness principles and practice on symptom change and findings suggest that MBIs may not be effective at targeting anxiety symptom severity.

### Effects as a Function of Intervention Type

Although there were no significant differences between MBCT and MBSR on primary symptom outcomes, subgroup analyses found that effects on primary symptom severity were significant for MBCT but not for MBSR. This replicates the finding by a previous meta-analysis [32] where a non-significant advantage for MBCT over MBSR was found. These findings, when taken in conjunction with the effects on depression outlined above, support the use of MBCT for people with a current diagnosis of a depressive disorder and extends the evidence for MBCT in depression, which was originally shown to be effective at reducing the risk of relapse for people who are in full or partial remission but who have experienced three or more episodes of depression [4], [5].

The single PBCT trial was not included in the moderator analysis however, it produced the largest effect size on depressive symptom severity (Hedges *g* = −1.81) for the studies of depressive disorders. Indeed, the effect size was more than twice as large as any other effect size in this particular analysis. The other trials in this analysis were of MBCT and it is of note that PBCT was originally developed for people currently experiencing severe mental health difficulties [52] and it may therefore be the case that PBCT lends itself well to people experiencing a current episode of depression. Mindfulness practices in PBCT are shorter (5 to 10 minutes) than in MBCT (up to 30 to 40 minutes) and frequent verbal guidance is given because of a concern that lengthier practices or extended periods of silence may be particularly challenging for people experiencing current distress [52]. Despite shorter mindfulness practices, findings from the current analysis show that the effect of PBCT on primary depressive symptom severity is not less than the effect of MBCT, although further studies of PBCT are required before drawing firm conclusions.

### Effects as a Function of Control Condition Type

Moderator analyses of control condition type showed that whilst effects of MBIs on symptom severity remained for studies using inactive control conditions (such as waiting lists), these effects disappeared when comparing MBIs to active control conditions. Four of the five studies which used an active control condition compared MBIs to group cognitive behaviour therapy (CBT). Two of these studies were for social anxiety disorder [44], [47], one was of MBCT for major depressive disorder [45] and one of MBSR for mixed anxiety disorders [39]. The mean effect size between MBIs and active control conditions on primary symptom severity was negligible (Hedges *g* = 0.03) which suggests that MBIs may be no less effective than group CBT. Whilst future non-inferiority studies are needed to compare MBIs with group CBT, particularly for the full range anxiety disorders, the evidence from the current meta-analysis shows promise for MBI as an alternative to group CBT.

### Attrition

A median of 15.5 percent of participants dropped out from the MBI conditions identified in this review. This is similar to the mean drop-out rate of 16.1 percent from a meta-analysis of RCTs of CBT [53]. This suggests that dropout rates from MBIs for people diagnosed with a current anxiety or depressive episode is not higher than would be expected from the broader psychotherapy literature. This is important as it suggests that engagement with MBIs is possible for people when they are experiencing a current episode of a depressive or anxiety disorder. Dropout rates were variable across the studies however, ranging from 8 percent to 37 percent and reasons for variability in dropout should be explored in future studies.

### Limitations

Research exploring the effectiveness of MBIs in populations with current diagnoses of anxiety or depression are in their infancy, and the evidence base is somewhat limited in both quantity and quality. Only 12 studies met our inclusion criteria, and of these the majority of studies (N = 7) compared MBI to inactive control conditions. These studies permit only a weak interpretation of the treatment effects, and do not allow the benefits of mindfulness practice and principles to be separated out from non-specific group therapeutic factors such as universality, altruism and group cohesion [54]. MBIs are purported to work through improving mindfulness which in turn is thought to reduce symptom severity and improve wellbeing. This is supported by evidence that improvements in mindfulness mediate symptom improvements following MBCT for recurrent depression [55]. In addition, a recent meta-analysis demonstrated enhanced mindfulness skills following MBIs in comparison to control conditions for people from non-clinical populations [17]. The preponderance of RCTs comparing to inactive control conditions limits the opportunities to explore mechanisms of change given the potential role of non-specific therapeutic factors in enhancing mindfulness and producing positive outcomes. Future research of MBIs should therefore aim to control for non-specific and non-mindfulness factors in order to isolate the potential benefits of learning mindfulness from other elements of MBIs.

The methodological quality of several of the included studies was poor, as shown by low Jadad ratings in some cases. Despite this, there were non-significant associations between Jadad ratings and effect sizes which indicates that there was not a bias towards lower quality trials reported larger effect sizes. Whilst seven of the 12 studies reported intention-to-treat data, five studies only reported completer data which potentially introduces some bias in favour of MBI, as it is possible that non-completers would have benefitted less than therapy completers. However this does not appear to be the case here as the mean effect size on primary symptom severity for the studies reporting completer data was similar to the effect size for studies reporting intention-to-treat data (Hedges *g* = −0.84 and −0.73 respectively).

Too few of the studies included long term follow-up of participants to allow for a separate analysis. For people experiencing a current episode of a depressive or anxiety disorder not only do we want our therapies to be of immediate benefit, we hope that they will continue to provide benefit in the longer term. Without following participants up it is not possible to know whether MBI provides long lasting benefit in relation to symptom reduction and future research in this area should include a follow-up period.

Whilst this meta-analysis set out to answer the question of efficacy of MBI for depressive and anxiety disorders, the range of anxiety disorders was not well represented. Whilst RCTs of social anxiety disorder [42], [44], [47], generalised anxiety disorder [40], health anxiety [46] and post-traumatic stress disorder [43] were included, no RCTs of MBIs specifically targeting obsessive compulsive disorder (see [56] for a recent review), agoraphobia, panic disorder or simple phobia could be found. This limits conclusions that can be drawn here about the effectiveness of MBIs transdiagnostically across anxiety conditions.

Efforts were made to limit the impact of publication bias on findings. Unpublished dissertations and theses were included in the search strategy and three of the major clinical trials registers were searched in order to find potential unpublished studies. No unpublished data were made available. Graphical (funnel plots) and statistical (Fail-Safe N) methods were used in order to assess for possible publication bias and its potential impact on findings. Whilst the funnel plot indicated a potential for publication bias (meaning that there may be unpublished trials with non-significant or negative findings), the Fail-Safe N analysis suggested that a large number of studies with nil effect would be needed to render the primary analysis non-significant. This allows us to have some confidence in our findings despite the omission of data from unpublished studies.

### Clinical Implications

This meta-analysis suggests that people meeting diagnostic criteria for a current episode of a depressive disorder can benefit from MBIs. However, the studies of MBIs for depression were limited to MBCT or PBCT; none of the studies were of MBSR. Therefore our findings only apply to MBCT and PBCT and not to MBSR and we cannot comment on the basis of our analysis on the effects of MBSR on current depression. Our findings suggest that people experiencing a current depressive episode can benefit from MBCT or PBCT despite the negative thoughts and feelings associated with depression, thinking processes that orient attention towards or away from negative content and motivational and attentional problems.

The studies targeting depression recruited from primary care and secondary care populations which suggests that MBIs might usefully be offered in both settings to people experiencing a depressive episode. It is of note however that the mindfulness-based intervention used in the PBCT trial [48] was especially adapted for secondary care populations [52]. None of the studies specifically recruited people from inpatient settings and therefore it would be premature to extend findings to this setting.

Finally, given the paucity of evidence in their favour, we would caution against offering MBIs as a first line intervention for people experiencing a primary anxiety disorder. Not only did we find few studies targeting specific anxiety disorders, but those we did find suggest that MBIs may not be effective at targeting primary symptom severity for people experiencing an anxiety disorder. There are other, well-evidenced interventions for the range of anxiety disorders [57], [58] and findings from the current meta-analysis would suggest great caution if offering MBIs to this population as a first line intervention instead of a well-established therapy.

### Conclusions

This is the first published meta-analysis of RCTs that evaluates the effectiveness of MBIs for people experiencing a current episode of a depressive or anxiety disorder. We found significant benefits relative to control conditions for primary symptom severity for people experiencing a current episode of depression following MBIs (namely MBCT or PBCT). Moreover, the analysis indicated that MBIs may produce similar outcomes to group CBT and therefore we suggest that MBCT or PBCT may be offered alongside other evidence-based interventions for people experiencing a current depressive episode in order to increase patient choice. We failed however to find support for MBIs for people experiencing a current episode of an anxiety disorder. This may well be due to the preponderance of small, underpowered studies but until further, adequately powered trials are conducted caution should be applied before offering MBIs as a first line intervention for anxiety disorders.

## Supporting Information

Checklist S1 **PRISMA Checklist.**
(DOC)
